# The comparison of plasma D-dimer levels in benign and malignant tumors of cervix, ovary and uterus

**Published:** 2015-07-01

**Authors:** Marzieh Vahid Dastjerdi, Soraya Ahmari, Sadaf Alipour, Afsaneh Tehranian

**Affiliations:** 1 Associated Professor, Department of Obstetrics and Gynecology, School of Medicine, Tehran University of Medical Sciences, Tehran, Iran; 2 Resident of Obstetrics and Gynecology, Department of Obstetrics and Gynecology, School of Medicine, Tehran University of Medical Sciences, Tehran, Iran; 3 Associated Professor, Surgery Department, Arash Women’s Hospital, Tehran University of Medical Sciences, Tehran, Iran; 4 Associated Professor, Department of Gynecology, Arash Hospital, Tehran University of Medical Sciences, Tehran, Iran

**Keywords:** D-dimer, Gynecologic Tumors, Tromboembolism, Coagulation

## Abstract

**Background:** Thromboembolism is the most important complication of cancers.The aim of this study was to determine D-dimer levels in benign and malignant tumors of the uterus, ovary and cervix.

**Subjects and Methods: **This was a cross sectional study and it was conducted on 90 female patients referred to Imam Khomeini and Arash Hospitals because of uterine, cervical and ovarian tumors in 2013-2014. After surgical resection or tissue biopsy, 2 cc of each patient’s blood was taken to be sent to laboratory of hospitals. “Nycocard” kit was chosen to measure D-dimer levels in Mg/Lit by neflumetry method. Data were analyzed in SPSS-16 by T-test and One-Way ANOVA test.

**Results: **The highest mean of D-dimer was 3.9 (± 2.9SD) in malignant cervical tumors. The mean plasma levels of D-dimer in malignant uterine cancers (P = 0.008), ovarian cancers (P = 0.007) and cervical cancers (P = 0.006) was significantly higher than benign tumors. In all three types of uterine, ovarian and cervical cancers, D-dimer was significantly higher in advanced stages than lower stages.

**Conclusion:** The plasma D-dimer levels in patients with malignant tumors of the uterus, cervix and ovary were higher than benign types. By increasing the stage of gynecologic malignant tumors, the levels of plasma D-dimer were increased.

## Introduction

 Endometrial cancer is the most common malignancy in women’s reproductive system and it is the cause of half of the gynecologic cancers in United States.^1^ Endometrial carcinoma is the fourth most common cancer in women (after breast, lung and colorectal cancers). It is also the eighth cause of female mortality due to malignancy.^2^

Cervical cancer is another common cancer in female and is the cause of 1.6% of death due to all malignancies in women and the cause of 15% of death due to all gynecologic cancers. It is the second cause of death for women between ages 20-39 after breast cancer.^3^ Ovarian cancer is the seventh common cancer in United States’ women, while it consist 5% of all female malignancies and is the most common cause of death due to gynecologic cancers in women.^4^ The prevalence of ovarian cancer is more in developed countries^5^ and more than two-third patients are in advanced stages when the disease is diagnosed.^6^

Cancer is a known cause of venous thromboembolism (VTE).^7^ The association between malignancy and activation of coagulation factors has been documented since many years ago. Thromboembolism is the most important side effect of cancer and second cause of death in cancer patients.^8^

The coagulopathy may be started directly by thrombin produced by tumor cells, or indirectly by stimulating of mononuclear cells or coagulation system.^9^ Recent studies showed that hyper coagulable state in cancer leads to poor prognosis of disease and cancer patients with VTE have less survival than the patients without VTE.^10^^, ^^11^

Some coagulation factors that show the effective role in tumor progression have been studied.^12^ One of the important factors is D-dimer and high D-dimer levels in cancer patients were associated with thromboembolism in a population based cohort study.^13^ In fact; high levels of D-dimer that indicate coagulation activity and fibrinolysis is reported in cancer patients in the absence of thrombosis. Studies showed that the high levels of D-dimer lead to poorer prognosis of tumor even without thromboembolism.^14^ Coagulation and fibrinolysis activity, as reflected by high plasma levels of D-dimer, is independently associated with poor prognosis in cancer and it is not necessarily mediated by the increased risk of VTE in patients with high D-dimer levels.^15^

Therefore, the elevated level of D-dimer may show progression of tumor and higher mortality.^16^ Though, a few studies addressed the differences between plasma D-dimer levels in different gynecologic tumors. The aim of this study is to determine and compare D-dimer levels as a tumor marker in patients with benign and malignant tumors of uterus, cervix and ovary.

## SUBJECTS AND METHODS

 The study was cross-sectional and population was the patients referred to Arash and Emam Khomeini hospitals with tumors of uterus, cervix and ovary between April 2013 to March 2014 for surgical resection or tissue biopsy. Inclusion criteria were patients suffered from uterine, cervical and ovarian cancer and the candidates for surgery and local biopsy. Exclusion criteria were contraindication of surgery and biopsy or failed biopsy and failure to obtain the informed consent. 15 patients were included in each subgroup of benign and malignant tumors by use of sample size formula (α= 5% and β =20%). 

 After surgical resection or tissue biopsy, 2 cc of each patient’s blood was taken to be sent to laboratory of hospitals. In laboratory, the blood sample was centrifuged for 15 minute and serum was separated from blood. The serum was then frozen and under freezing chain conditions was transferred to another laboratory to determine the level of D-dimer. “Nycocard” was chosen to measure D-dimer levels (in Mg/Lit) by Neflumetry method. The levels that were higher than 0.3 Mg/Lit were considered as high levels and the measures below it were considered as low levels of D-dimer. It should be noted that the accuracy of kit was 0.1-20 Mg/Lit. The results of tumor surgery and local biopsy were collected by researcher, as well and verification of the tumors was performed by a pathologist. The analysis was processed using the Statistical Package for Social Sciences (SPSS), version 16. Data were represented as mean and standard deviation and two way tables. The mean differences between D-dimer levels in benign and malignant tumors were analyzed using T-test and One-Way ANOVA test. In all cases the results were statistically significant when p-values reported less than 0.05 (P<0.05).

## Results

 The study was conducted on 90 female patients who were diagnosed with benign and malignant uterine, cervical and ovarian tumors and were divided in 6 subgroups of 15 patients. Mean ages of patients with benign tumors was 40.7 (± 4.6SD) and the ones with malignant tumors was 51(± 3.6SD). The mean level of D-dimer was the highest in malignant cervical tumors (3.9 Mg/Lit) and it was the lowest in benign uterine tumors (0.27 Mg/Lit). The mean plasma level of D-dimer in benign and malignant tumors of uterus (P=0.008), cervix (P=0.006) and ovary (P=0.007) was significantly different and it is higher in malignant tumors. The characteristics of patients’ malignant and benign tumors are available in Table 1.

Then, D-dimer level was evaluated in malignant gynecologic cancers. Among malignancies, the level of D-dimer was significantly higher in cervical cancer than uterine and ovarian cancer (P<0.05). The characteristics of patients’ malignant tumors are available in Table 2. In uterine cancer, the level of D-dimer was significantly different in various stages and the highest difference was between stage 1 and 4 (P=0.002).

**Table 1:** The characteristics of patients’ malignant and benign tumors of uterus, cervix and ovary

**Tumors**	**Type**	**Number**	**Mean**	**Std. Deviation**
**Uterine **	benign	15	0.27	0.281
**Cancer**	malignancy	15	1.61	1.797
**Ovarian **	benign	15	0.37	0.589
**Cancer**	malignancy	15	2.54	2.805
**Cervical **	benign	15	1.18	1.955
**Cancer**	malignancy	15	3.90	2.994

In ovarian malignancies, significant different levels of D-dimer were observed between stages either 1 and 4, and 2 and 4 and the more stages cancer proceeds, the higher the level of D-dimer would be. In malignant cervical tumors, the differences of levels of D-dimer between various stages weren’t significant, but stages 1 and 4had significantly different levels of D-dimer, when post hoc test was LSD (P= 0.045).

## Discussion

 The purpose of the study was to compare D -dimer levels in patients with benign and malignant tumors of uterus, cervix and ovary. The study indicated that plasma levels of D-dimer increased in malignant tumors. The mean level of D-dimer was significantly higher in malignant cervical tumors than uterine and ovarian tumors. Also, in all types of uterine, cervical and ovarian cancers, the level of D-dimer in advanced stages was significantly higher than the lower stages. In present study, D-dimer which shows the activity of fibrinolytic system in coagulation process was used as a marker to evaluate the relationship between abnormal coagulation/fibrinolysis and progression of gynecologic cancers. Several tumors are investigated about the level of D-dimers and their prognosis. Most tumors that have been studied are lung,^17^ colon,^18^ prostate^19^ and breast^20^ tumors and this is probably because of their higher prevalence in different population. 

**Table 2:** The characteristics of patients’ malignant tumors of uterus, cervix and ovary

**Malignancy **	**STAGE**	**NO**	**Mean**	**Std. Deviation**
**Uterus**	1	5	0.26	.089
	2	0	0	0
	3	8	1.68	1.298
	4	2	4.70	2.263
**Ovary**	1	3	0.10	0.000
	2	3	0.17	0.115
	3	6	3.08	2.472
	4	3	6.27	0.839
**Cervix**	1	2	0.10	0.000
	2	5	3.66	2.887
	3	4	4.48	2.427
	4	4	5.53	3.337

The mean level of D-dimer was the highest in malignant cervical tumors and it may refer to the different mechanism of VTE in gynecologic tumors. Previous studies showed that the level of D-dimer will increase by progression of ovarian cancer to higher stages.^21^^,^^22^

Rose et al. showed that levels of D-dimer were correlate with tumor markers such as CA-125 in ovarian cancer.^23^ Yousef et al. also indicated that tissue factor and other coagulation components that generate local thrombin can be increased in ovarian cancer.^24^

The mechanism of thrombosis is different in uterine cancer and Polterauer et al. reported that higher production of plasma fibrinogen can cause thrombosis which is related to tumor progression.^25^ other studies have proved the production of fibrinogen by uterine tumors.^26^^,^^27^ The level of fibrinogen in plasma affects not only the local progression of uterine tumors but also its metastasis.^28^^,^^29^ In uterine cancer, metastatic disease, extra uterine spread or FIGO stage III/IV and D-dimer more than 1.5 Mg/ml before treatment are the risk factors of thromboembolism at initiation of treatment.^30^ In Boinget et al. study*,* the presence of metastatic tumors is a strong determinant of thrombosis in uterine cancer, and in metastasis the probability of thrombosis multiplied by 6.4 in comparison to local tumor.^31^

In cervical tumors the mechanism of VTE is different and thrombocytosis strongly affects survival of patients, even after adjustment were made for stage, histological type and age.^32^ If the count of platelets increases by more than 400000, the relative risk of cancer death will be multiplied by 1.5 in cervical cancer.^33^

Increasing of the level of D-dimer and its relationship with malignancy of gynecologic tumors is important due to different aspects. First, it defines the necessity of preventive treatment of thrombosis in gynecologic cancers. Second, it can be used as a marker beside other tumor markers for better determination of tumor progression. It seems that more studies are required to investigate the use of coagulation markers for defining the progression of gynecologic tumors.

It’s suggested to design the study to follow patients, so the relationship between prognosis of disease or the stages of tumors and the level of plasma D-dimer can be exactly investigated.

Increasing the sample size can lead to more accurate evaluation of plasma D-dimer levels in different stages of gynecologic cancers in next studies. 

## CONCLUSION

 Increasing of the level of D-dimer and its relationship with malignancy of gynecologic tumors is important due to different aspects. First, it defines the necessity of preventive treatment of thrombosis in gynecologic cancers. Second, it can be used as a marker beside other tumor markers for better determination of tumor progression. It seems that more studies are required to investigate the use of coagulation markers for defining the progression of gynecologic tumors.

It’s suggested to design a study to follow patients, so the relationship between prognosis of disease and the plasma level of D-dimer can be exactly investigated. Increasing the sample size can lead to more accurate evaluation of level of D-dimer in different stages of gynecologic cancers in next researches. 
